# Modified Hyperspheres Algorithm to Trace Homotopy Curves of Nonlinear Circuits Composed by Piecewise Linear Modelled Devices

**DOI:** 10.1155/2014/938598

**Published:** 2014-08-11

**Authors:** H. Vazquez-Leal, V. M. Jimenez-Fernandez, B. Benhammouda, U. Filobello-Nino, A. Sarmiento-Reyes, A. Ramirez-Pinero, A. Marin-Hernandez, J. Huerta-Chua

**Affiliations:** ^1^Electronic Instrumentation and Atmospheric Sciences School, Universidad Veracruzana, Cto. Gonzalo Aguirre Beltrán S/N, 91000 Xalapa, VER, Mexico; ^2^Higher Colleges of Technology, Abu Dhabi Men's College, P.O. Box 25035 Abu Dhabi, UAE; ^3^National Institute for Astrophysics, Optics and Electronics, Luis Enrique Erro No. 1, Santa María Tonantzintla, 72840 Puebla, PUE, Mexico; ^4^Department of Artificial Intelligence, Universidad Veracruzana, Sebastián Camacho No. 5, 91000 Xalapa, VER, Mexico; ^5^Facultad de Ingeniería Civil, Universidad Veracruzana, Venustiano Carranza S/N, Colonia Revolución, 93390 Poza Rica, VER, Mexico

## Abstract

We present a homotopy continuation method (HCM) for finding multiple operating points of nonlinear circuits composed of devices modelled by using piecewise linear (PWL) representations. We propose an adaptation of the modified spheres path tracking algorithm to trace the homotopy trajectories of PWL circuits. In order to assess the benefits of this proposal, four nonlinear circuits composed of piecewise linear modelled devices are analysed to determine their multiple operating points. The results show that HCM can find multiple solutions within a single homotopy trajectory. Furthermore, we take advantage of the fact that homotopy trajectories are PWL curves meant to replace the multidimensional interpolation and fine tuning stages of the path tracking algorithm with a simple and highly accurate procedure based on the parametric straight line equation.

## 1. Introduction

The circuit simulation tools are constantly improved in order to cope with the challenges due to the new fabrication technologies. Among the circuit analysis methodologies, the direct current (DC) analysis is highlighted as one of the most important because it describes the static behaviour of the circuits. As a result of the DC analysis of nonlinear circuits, one obtains a nonlinear algebraic equations system (NAES). The most common method applied to solve such equations is the Newton-Raphson method (NRM). However, it is common that NRM fails due to its well-known problems of convergence: oscillation and divergence to infinity, among others. In fact, NRM has a local convergence only, which means that if the starting point is not close enough to the sought solution the method will probably diverge. What is more, if the circuit under analysis is multistable, then NRM will not be helpful because it can locate only one solution per simulation, ignoring the existence of more solutions. Therefore, the homotopy continuation method (HCM) [1–37] arises as an alternative to NRM due to its characteristics: to find multiple operating points and better convergence [38].

In recent years, the PWL modelling technique gained popularity as a tool for circuit simulation and other related areas [39, 40]. The basic idea is to replace traditional models by their piecewise linear (PWL) representations [41–44]. The main advantages are reduction of equations complexity, the straightforward inclusion of empirical models, and potential replacing of piecewise models by their unified PWL representation. Several methodologies have been proposed to find multiple solutions of PWL circuits [45–57].

However such methodologies exhibit some drawbacks like the requirement of several initial points to find multiple solutions [53, 54], the use of implicit PWL models [55, 56], and the need of expressing the circuit equations in terms of the linear complementary problem (LCP) that implies computing model state variables [57]. Therefore, in order to circumvent the aforementioned disadvantages, we explore the application of HCM methods in combination with an adaptation of the modified spheres algorithm (MSA) [37] for the DC analysis of PWL circuits.

This paper is organized as follows. A brief description of PWL modelling is presented in Section 2. In Section 3, we introduce the proposed HCM and its path following technique (MSA). In Section 4, four case studies of nonlinear circuits are presented and solved by using a HCM method. Numerical simulations and a discussion about the results are provided in Section 5. Finally, a concluding remark is given in Section 6.

## 2. Brief Description of PWL Modelling

A mathematical model approach, widely used in nonlinear circuit analysis, is the so-called piecewise linear (PWL). The aim of this kind of modeling is to approximate the nonlinear behavior of a circuit element by using a set of linear mappings. This means transforming a single nonlinear equation into a finite number of linear equations. One of the first piecewise linear models was provided by Chua and Kang in [58]. Another proposal was presented by Van Bokhoven in [59]. Subsequent contributions were the extension of the Chua model reported by Guzelis and Goknar in [60] and the parametric proposal given by Vandenberghe et al. in [61], among others. While there are diverse proposals of PWL models, they can be classified into two classes. The first one contains explicit models. For this class of models, the output vector can be obtained by just substituting the input vector into the model. The second one contains models which are implicit. In such models the output vector cannot be obtained directly. In contrast, an algorithm has to be performed by which the output vector is computed [62]. The more representative examples of explicit and implicit PWL descriptions are the canonical model of Chua1 and the model of Bokhoven1, respectively.

The formal definition of the Chua1 model is expressed as follows.

**Theorem 1 .**

Any one-dimensional piecewise linear curve with *L* segments and *σ* break points *β*_1_ < *β*_2_ < ⋯<*β*_*σ*_ can be represented by the expression

(1) $$\begin{array}{c} y\left(x\right)=a+bx+\overset{\sigma }{\underset{i=1}{\sum }}c_{i}\left|x-\beta_{i}\right|, \end{array}$$

where the model parameters can be computed by

(2) $$\begin{array}{c} a=y\left(0\right)-\overset{\sigma }{\underset{i=1}{\sum }}c_{i}\left|\beta_{i}\right|,\\ b=\frac{J^{\left(1\right)}+J^{\left(\sigma +1\right)}}{2},\\ c_{i}=\frac{J^{\left(i+1\right)}-J^{\left(i\right)}}{2},\;i=1,2,\ldots ,\sigma , \end{array}$$

with *J* ^(*i*)^ denoting the slope of the *i*th constitutive segment in the piecewise linear curve.

Meanwhile, the Bokhoven1 model is expressed by a state variable system defined in formulation of LCP. For further details about LCP, the reader is referred to [63].

The main factor that motivates the use of PWL models is the simplicity of their structure, which is linear in each region of the domain. However, in terms of circuit analysis the use of piecewise linear models means transforming a single nonlinear equation into several linear equations that could easily be solved by standard methods from linear algebra. The problem lies now in the extremely large number of linear regions to be discarded to determine the entire set of circuit solutions. Unfortunately, this task requires enormous computational resources. To overcome that problem several methodologies and algorithms have been proposed. For example, Chua and Ying [64] reported an efficient method where the number of linear simultaneous equations to be solved could be decreased by a sign test. The same idea is improved by Yamamura and Ochiai in [65] where linear programming techniques are applied and a more efficient sign test algorithm is also reported. Katzenelson presents an algorithm based on Newton's homotopy in [53], and more recently Tadeusiewicz and Kuczyński offered a method that combines the homotopy concept and the theory known as a linear complementary problem [57].

## 3. The Proposed Homotopy Scheme

The equilibrium equation to describe the DC behaviour is obtained using the Kirchhoff laws, resulting in

(3) $$\begin{array}{c} f\left(x\right)=0,\;f\in R^{n}⟶R^{n}, \end{array}$$

where **x** represents the electrical variables of the circuit and *n* the number of variables.

Homotopy methods are based on the fact that solutions are connected by a curve denominated “solution curve” or “homotopy curve.” Such curve is induced by including an extra parameter in the original NAES, resulting in

(4) $$\begin{array}{c} H\left(f\left(x\right),\lambda \right)=0,\;H\in R^{n}\times R⟶R^{n}, \end{array}$$

where *λ* is the homotopy parameter and **H** ^−1^(0) the family of solutions that conforms the homotopy path.

An example of homotopy formulation is Newton's homotopy

(5) $$\begin{array}{c} H\left(f\left(x\right),\lambda \right)=f\left(x\right)+\left(\lambda -1\right)f\left(x_{i}\right)=0, \end{array}$$

where **x**_*i*_ is the starting point of the trajectory.

This system has the following properties.

(1) At the starting point *λ* = 0,
  (6) $$\begin{array}{c} H\left(f\left(x\right),0\right)=f\left(x\right)-f\left(x_{i}\right)=0, \end{array}$$
  where the homotopy system admits at least the solution **x**_*i*_.
(2) The deformation continues until crossing *λ* = 1 where
  (7) $$\begin{array}{c} H\left(f\left(x\right),1\right)=f\left(x\right)=0; \end{array}$$
  that is, the homotopy is reduced to (3).

Thus, the original problem becomes a numerical continuation problem [4, 5, 12, 13, 21, 25–28], where the continuation variable is the homotopy parameter *λ*. The homotopy map creates a continuous line that crosses several times *λ* = 1 depending on the number of operating points. A drawback of the homotopy methods is that there is no generalized methodology to guarantee that a single homotopy path possesses all the operating points of any given nonlinear circuit. In contrast, HCM can locate multiple operating points in comparison to NRM that can fail to find even a single operating point.

### 3.1. Modified Spheres Algorithm

Once the equilibrium equation and homotopy map are constructed, a new problem emerges: the homotopy trajectory should be traced in order to detect the roots. It is well known from the literature that if the path tracking algorithm is not correctly implemented, the simulation may fail to detect any root even though the roots are, in fact, along the curve [4, 5, 12, 13, 21, 25–28]. For the case of PWL circuits, the problem for the path tracking algorithm lies in the prediction stage, because most of the predictor mechanisms are based on the tangent of the homotopy curve. If we consider that the derivative of PWL functions is not defined at the break points, then the tangent of the homotopy curve can not be evaluated at such points. Therefore, we propose adapting the modified spheres algorithm (MSA) for the path following of the homotopy curves of PWL circuits, which is not based on the use of tangents of the trajectory.

The homotopy formulation contains *n* equations and (*n* + 1) variables, where *x*_*i*_  (*i* = 1,…, *n*) represent the variables of the system and *x*_*n*+1_ is the homotopy parameter *λ*. Nevertheless, if we add the equation that describes a sphere [2, 3, 13, 37, 66] with center at *c* (initial point of the trajectory) and radius *r* expressed by

(8) S(x1,x2,…,xn+1)=(x1−c1)2+(x2−c2)2 +⋯+(xn+1−cn+1)2−r2=0,

then, it is possible to apply a regular NRM to solve the homotopy formulation.

Therefore, using (4) and (8), we formulate the augmented system as

(9) H1(f1(x),λ)=0,H2(f2(x),λ)=0,⋮Hn(fn(x),λ)=0,S(x1,x2,…,xn,λ)=0.

**Figure 1:**
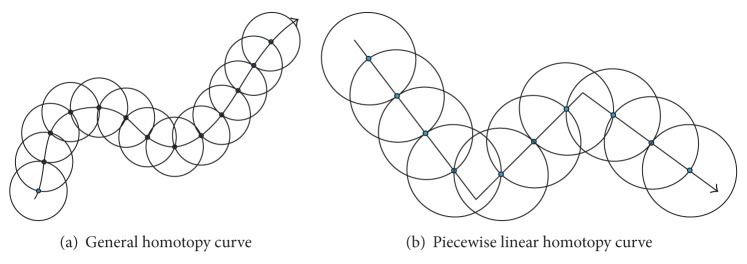
Solution curves with spheres [37].

The solution curve can be traced by solving (9) for each hypersphere and updating the center of the hypersphere in each iteration step. The hyperspheres (*S*_1_, *S*_2_,…) are allocated successively as shown in Figure 1(a); at each step the solution obtained is used as the center of the new sphere. In the same fashion, Figure 1(b) depicts the application of MSA algorithm for the path tracking of PWL curves.

The proposed adaptation of the MSA scheme [37] for the Newton homotopy applied to PWL circuits is described as follows.

**Figure 2:**
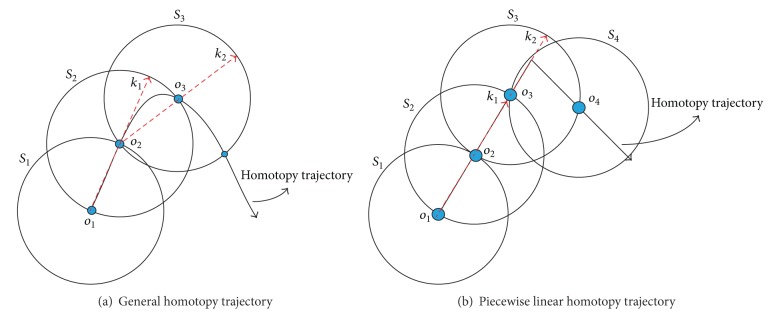
Spheres algorithm [37].

**Figure 3:**
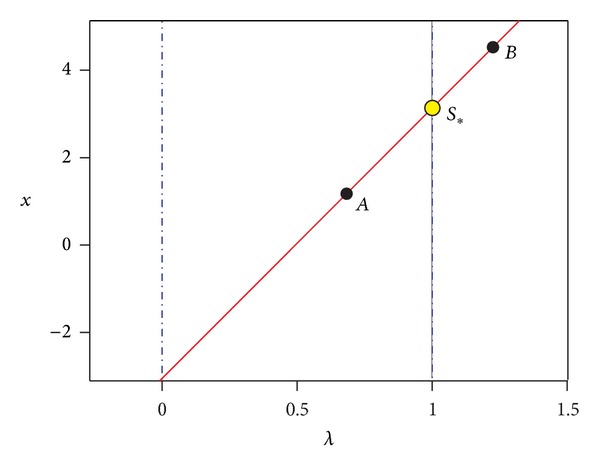
Interpolation procedure using a parametric straight line.

(i) Predictor: we use points *O*_1_ and *O*_2_ to predict the point *k*_1_. The next predictor point and successive points are obtained as depicted in Figure 2(a).
(ii) Corrector: after calculating the point predictor (*k*_1_), a corrected point (*O*_3_) is calculated by solving (9). This procedure is detailed in [37]. Nonetheless, if we consider that—for this work—the homotopy trajectory is described as a PWL curve, then the corrector step will require most of the time one iteration to correct the prediction over straight lines, except at the break points, where it will require more steps to correct the curve (see Figure 2(b)).
(iii) There is a potential issue called reversion phenomenon that provokes a backward tracing. In [37] a strategy based on gradients and angles of the intersection of the sphere along the trajectory is proposed.
(iv) Find zero strategy [12, 22]: the finding zero strategy should start after the trajectory crosses *λ* = 1. This procedure requires detecting the two points (*A* and* B*) before and after *λ* = 1 as depicted in Figure 3.
(v) Interpolation of operating points [12, 22]: traditional schemes of path tracking algorithms require the application of complicated multidimensional interpolation algorithms as those reported in [37]. Nonetheless, as we will show in the cases study section, the homotopy trajectory of PWL circuits is also a PWL curve. Therefore, we propose using the formula of a parametric straight line to interpolate the solution at *λ* = 1. Using the points *A* and *B*, we create two vectors **A** and **B**, respectively, resulting in the following equation:
  (10) $$\begin{array}{c} B+t\left(B-A\right)=0, \end{array}$$
  where *t* is the parameter that describes the *n* + 1-dimensional straight line. To perform the interpolation, we obtain the value of *t* that induces *λ* = 1 and update the rest of the equations to obtain the sought solution *S*_∗_ (see Figure 3). This process can be repeated each time the homotopy trajectory crosses *λ* = 1.
(vi) Improving accuracy for final solutions also known as fine tuning [22]: traditional path following schemes including the ones reported for the MSA scheme [13, 37] require extra steps of NRM to improve the accuracy of the interpolated solutions. However, the aforementioned interpolation step can theoretically obtain a highly accurate solution. The reason relies on the fact that the homotopy curve crosses exactly over the roots of the equilibrium equation; then, the straight line (10) also crosses over the exact solution.

## 4. Cases Study

In the present section, we will solve four case studies [64] to show the usefulness of the proposed method to perform the DC analysis of nonlinear circuits composed of devices modelled using the explicit PWL model (1). For all the cases' study, we use a constant radius *r* = 0.1 for the hyperspheres.

### 4.1. Circuit with Two Nonlinear Resistors

**Figure 4:**
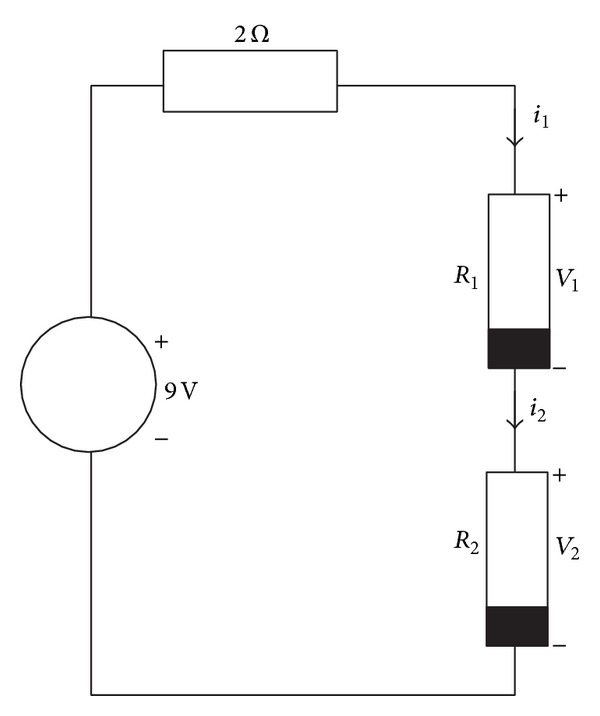
Two nonlinear resistor circuits.

The following case study shows a simple circuit composed of two nonlinear resistors as depicted in Figure 4. The models of the resistors *R*_1_ and *R*_2_ are

(11) R1:i1=−1258+98v1+78|v1+1|−32|v1−2|+34|v1−5|−18|v1−11|−98|v1−13|+2|v1−15|,R2:i2=294+32v2−32|v2+8|+32|v2+5|−32|v2+3|+32|v2+1|−34|v2−3|−54|v2−8|+32|v2−10|+|v2−13|−54|v2−16|+14|v2−18|,

described by 7 and 11 PWL segments, respectively.

Using Kirchhoff laws, we obtain

(12) f1(v1,v2)=v1+v2+2i1−9=0,f2(v1,v2)=v1+v2+2i2−9=0.

Applying the Newton homotopy to (12) combined with MSA yields

(13) H1(v1,v2,λ)=f1(v1,v2)+(λ−1)f1(v1,0,v2,0)=0,H2(v1,v2,λ)=f2(v1,v2)+(λ−1)f2(v1,0,v2,0)=0,S(v1,v2,λ)=(v1−c1)2+(v2−c2)2+(λ−c3)2−r2=0,

where *v*_1,0_ = −5 and *v*_2,0_ = −4 are the initial point of the homotopy at *λ*_0_ = 0 and *S*(*v*_1_, *v*_2_, *λ*) is the equation of the hypersphere whose center will be updated at each iteration of the method.

**Figure 5:**
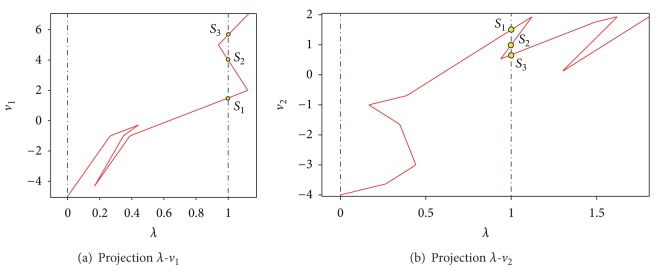
Homotopy path for (13).

For the first hypersphere the center is located at *c*_1_ = *v*_1,0_, *c*_2_ = *v*_2,0_, and *c*_3_ = *λ*_0_. The centers of the successive hyperspheres are obtained using the aforementioned procedure in Section 3.1. As a result of MSA algorithm, the three operating points of the circuit have been located (see Figure 5). In addition, Table 1 shows the computed solutions, iterations, and the mean square error (MSE).

### 4.2. Circuit with Three Nonlinear Resistors

**Figure 6:**
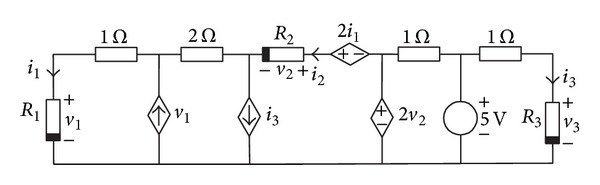
Three nonlinear resistor circuits.

The following case study shows a circuit composed of three nonlinear resistors as depicted in Figure 6. The models of *R*_1_, *R*_2_, and *R*_3_ resistors are

(14) R1:i1=56|v1+6|−56|v1−6|,R2:v2=16|i2+1|−16|i2−5|,R3:i3=v3−54|v3−1|+2|v3−2|−|v3−3|,

described by 3, 3, and 4 PWL segments, respectively.

Using Kirchhoff laws [64], we obtain

(15) f1(v1,v2,v3)=v1+i2+v3−i1−5=0,f2(v1,v2,v3)=i2+v3−v2−5=0,f3(v1,v2,v3)=−v3−i3+5=0.

**Figure 7:**
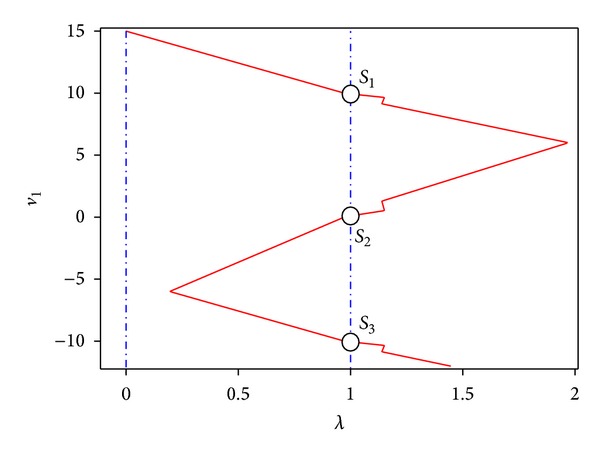
Projection *λ*-*v*_1_ of homotopy path for (15).

Next, we apply the Newton homotopy to (15) as done for the first case study, using *v*_1,0_ = 15, *i*_2,0_ = −1, and *v*_3,0_ = 15 as the initial point of the homotopy. As a result of tracing the homotopy path, the three operating points of the circuit have been located (see Figure 7). In addition, Table 2 shows the found solutions, iterations, and the mean square error (MSE).

### 4.3. Schmitt Trigger Circuit

**Figure 8:**
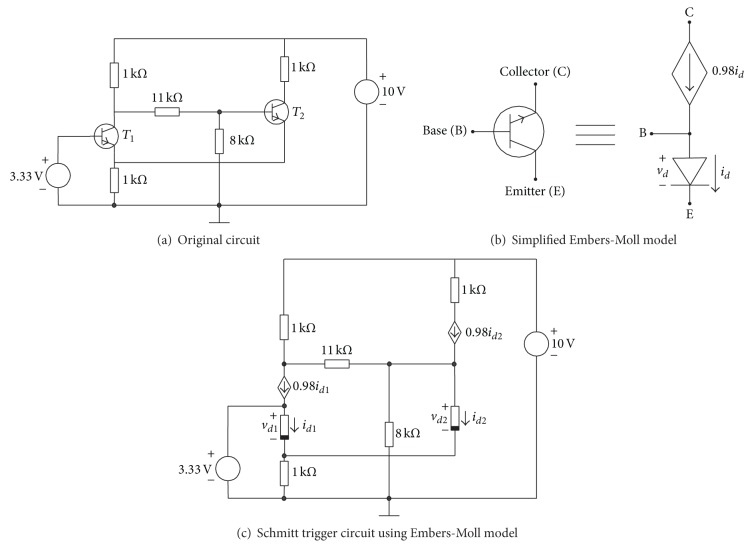
Schmitt trigger circuit.

Consider the Schmitt trigger circuit of Figure 8(a), where the bipolar transistors are modelled using the simplified Ebers-Moll (see Figure 8(b)) model of NPN transistors as depicted in Figure 8(c). The PWL model of five segments of the diodes of all transistors is

(16) id(vd)=−0.05486777833+0.1482755558vd+0.01157779318|vd−0.306|+0.01181869788|vd−0.3375|+0.04904536922|vd−0.366|+0.07583369515|vd−0.3875|.

Using Kirchhoff laws, we obtain

(17) f1(v1,v2)=3.33−1000(id(v1)+id(v2))−v1=0,f2(v1,v2)=4−1392id(v1)−1096id(v2)−v2=0.

**Figure 9:**
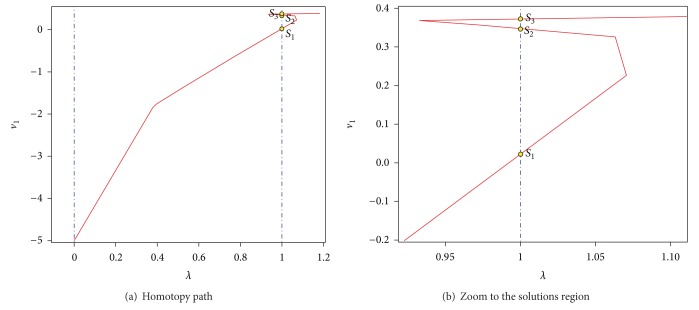
Homotopy path for (17) projected over *λ*-*v*_1_.

Then, Newton homotopy is applied in the same fashion as in the first example, using as starting point *v*_1,0_ = −5 and *v*_2,0_ = −2 at *λ* = 0. The results show that the homotopy trajectory crosses for the three operating points of the Schmitt trigger circuit as depicted in Figure 9 and Table 3.

### 4.4. Chua's Circuit with Nine Solutions

**Figure 10:**
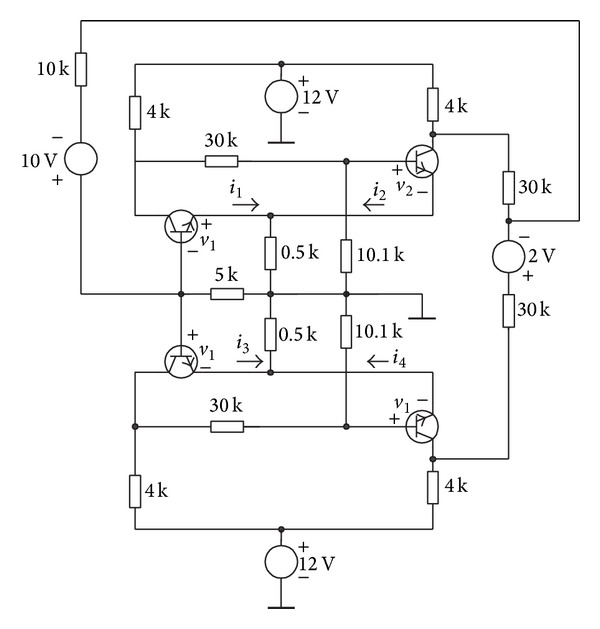
Chua's circuit with nine solutions.

Consider Chua's circuit of Figure 10, where the bipolar transistors are modelled using the simplified Ebers-Moll (see Figure 8(b)) model of NPN transistors. The PWL model for the diodes of all transistors is (16).

Using Kirchhoff laws, we obtain

(18) f1(v1,v2,v3,v4)=4.36634v2+6103.168id(v1)+2863.168id(v2)−12=0,f2(v1,v2,v3,v4)=5.4v1+v3+3580id(v1)+6620id(v2)+700id(v3)+500id(v4)−22=0,f3(v1,v2,v3,v4)=4.36634v4+6103.168id(v3)+2863.168id(v4)−12=0,f4(v1,v2,v3,v4)=v1+5.4v3+700id(v1)+3580id(v3)+6620id(v4)−22=0.

**Figure 11:**
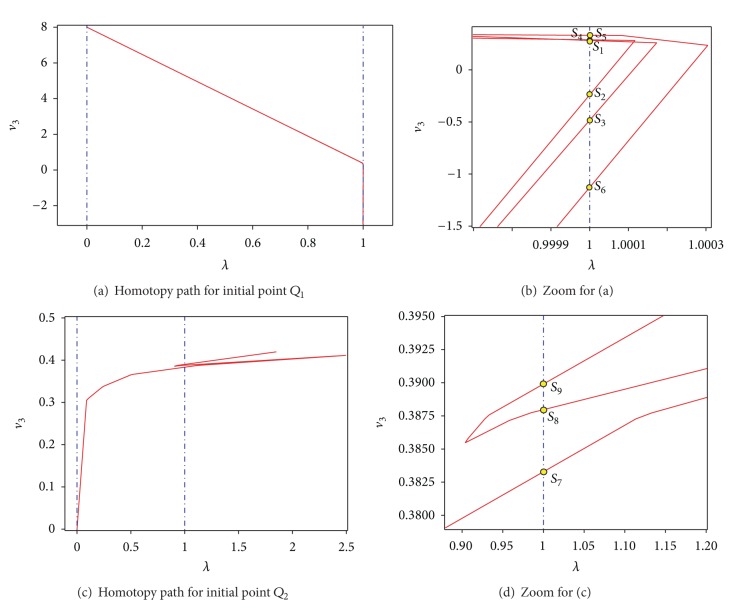
Homotopy paths for (18) projected over *λ*-*v*_3_.

The Newton homotopy is applied to (18) in the same way as in the first case study. We trace two trajectories with the following starting points: *Q*_1_ = [−7, −1,8, 1] and *Q*_2_ = [0, −7,0, 0]. After using the adapted MSA algorithm, the nine solutions of the circuit were found (see Figure 11). In addition, Table 4 shows the found solutions, iterations, and the mean square error (MSE).

## 5. Numerical Simulation and Discussion

All case studies were successfully solved using the proposed methodology. For the first three case studies, it was possible to find within a single trajectory the three operating points of each problem, and for the last case study, we find the nine solutions of Chua's circuit using two starting points. The high accuracy of the located operating points shows that the simple interpolation algorithm based on straight lines is a powerful tool and is simple to implement (see Tables 1–4). Besides, the accuracy of the interpolate solutions allows us to discard the stage of applying NRM extra steps to increase accuracy usually required by path tracking algorithms [4, 5, 12, 13, 21, 25–28]. It is important to remark that the variety of solved circuits exhibits the high potential of HCM combined with MSA to solve multistable nonlinear circuits integrated by devices modelled with explicit PWL representations.

In [53, 54] methods based on the Newton homotopy are reported, which are capable of locating only one solution per simulation. Therefore if user requires to find more solutions, it is necessary to propose some random initial points to perform more simulations. Instead, the proposed methodology is capable of locating multiple operating points within a single path or simulation.

Methods reported in [55, 56] use implicit PWL models. This implies that the number of linear regions explodes due to the diode synthesis. Besides, compared to the explicit models, implicit PWL models require a more complex algorithm to compute the model state variables. The proposed methodology uses an explicit model representation easy to implement.

In [57] a methodology that depends on the specific circuit topology description of multiport with extracted ideal diodes is reported. In such methodology, circuit equations are expressed in terms of the LCP which implies computing model state variables. The proposed methodology is based on a straightforward methodology based on the traditional circuit analysis tools used to build commercial circuit simulators and a simple path tracking algorithm easy to implement.

Further research should be addressed in the following topics.

- Implement a strategy to use the fact that the homotopy curves are straight lines to accelerate the homotopy simulation.
- Implement a circuit simulator to solve high density transistor circuits modelled by the PWL technique.
- Replace the Newton homotopy by other methods as the fixed point homotopy [14], double bounded homotopy [12, 37], double bounded polynomial homotopy [11, 36], Newton fixed-point homotopy [67], *d*-homotopy [68], and multiparameter homotopy [13, 17], among others. This research can lead to proposal of better homotopy schemes with better results in aspects like number of found solutions, CPU time, and global convergence, among others.
- Theoretically obtain the position of the break points of the PWL homotopy curve, significantly decreasing the number of steps. Such research can conduct to a very fast path tracking scheme.
- Propose a methodology to obtain an optimal initial point for the homotopy simulation. This golden starting point will possess the characteristic of producing a minimum number of iterations and a maximum number of found solutions or all solutions.

## 6. Conclusions

In this work, we presented a homotopy scheme based on the Newton homotopy and a modified MSA path tracking algorithm, applied to the DC simulation of nonlinear circuits composed of devices modelled by PWL techniques. The effectiveness and power of the proposed scheme were exhibited by the successful solution of all the operating points of several circuits including devices as nonlinear resistors, diodes, transistors, and transactors, among others. In addition, the high accuracy of the solutions was reached by applying a simple interpolation technique that discards the use of Newton-Raphson extra steps to increase the accuracy of the interpolated solutions. Finally, further research should be performed to extend the application of the proposed scheme to very large integrated circuits (VLSI).

## Figures and Tables

**Table 1:** Numerical solutions for (12).

Solution	Iteration	*v*_1_	*v*_2_	MSE = (*f*_1_^2^ + *f*_2_^2^)/2
*S*_1_	168	1.49999999999	1.49999999998	0
*S*_2_	196	4.00000000122	0.999999999476	2.22*e* − 18
*S*_3_	214	5.66666666670	0.666666666664	2.12*e* − 20

**Table 2:** Numerical solutions for (15).

Solution	Iteration	*v*_1_	*i*_2_	*v*_3_	MSE
*S*_1_	137	9.9333333333	2.20000000000	2.86666666667	1.71*e* − 22
*S*_2_	382	0.0999999999	2.20000000000	2.86666666666	1.45*e* − 22
*S*_3_	610	−10.0666666666	2.20000000000	2.86666666667	3.86*e* − 23

**Table 3:** Numerical solutions for (17).

Solution	Iteration	*v*_1_	*v*_2_	MSE
*S*_1_	59	0.022145787125	0.374592098924	6.11*e* − 18
*S*_2_	63	0.348923957274	0.358608185920	1.70*e* − 21
*S*_3_	68	0.372176159314	−0.117291118129	5*e* − 21

**Table 4:** Numerical solutions for (18).

Path	Solution	Iteration	*v*_1_	*v*_2_	*v*_3_	*v*_4_	MSE
*Q*_1_	*S*_1_	105	−0.734973033383	0.376723496304	0.318166142629	0.372621919721	1.14*e* − 17
*Q*_1_	*S*_2_	111	−0.650249656974	0.376723495848	−0.239265377696	0.376723494677	6.64*e* − 14
*Q*_1_	*S*_3_	235	0.326931131382	0.369666979551	−0.48305281023	0.376723495005	3.61*e* − 16
*Q*_1_	*S*_4_	243	0.329725453371	0.368724929946	0.324331786706	0.370543296483	6.27*e* − 19
*Q*_1_	*S*_5_	312	0.386520101863	−4.29094430670	0.337714498625	0.365872023364	6.40*e* − 17
*Q*_1_	*S*_6_	328	0.388146243604	−4.75726420472	−1.12762657849	0.376723498380	5.69*e* − 17
*Q*_2_	*S*_7_	58	0.383283219902	−3.63542706051	0.383283217035	−3.63542647848	1.73*e* − 16
*Q*_2_	*S*_8_	193	0.338139358469	0.364994969864	0.387969158453	−4.68386026807	2.86*e* − 19
*Q*_2_	*S*_9_	215	−1.19554608083	0.376723498781	0.389904592568	−5.48612114477	2.42*e* − 16
